# Circulating kidney injury molecule-1 is a novel diagnostic biomarker for renal dysfunction during long-term adefovir therapy in chronic hepatitis B

**DOI:** 10.1097/MD.0000000000005264

**Published:** 2016-11-04

**Authors:** Ziyue Li, Chuan Shen, Yadong Wang, Wei Wang, Qian Zhao, Zhenzhong Liu, Yang Wang, Caiyan Zhao

**Affiliations:** a Department of Infectious Disease, The Third Affiliated Hospital of Hebei Medical University; b Department of Liver Disease, The Fifth Hospital of Shijiazhuang, Shijiazhuang, China.

**Keywords:** adefovir dipivoxil, entecavir, hepatitis B virus, kidney injury molecule-1, renal impairment

## Abstract

The aim of this study was to evaluate serum kidney injury molecule-1 (KIM-1) as a new diagnostic marker of renal dysfunction in chronic hepatitis B (CHB) patients receiving long-term adefovir dipivoxil (ADV) treatment.

We retrospectively enrolled 85 patients treated with ADV and 85 patients treated with entecavir (ETV) monotherapy, for at least 6 months. The 2 groups were matched for baseline age (± 5 years), sex, and estimated glomerular filtration rate (eGFR). Serum creatinine, cystatin C, and KIM-1 concentrations were measured, and eGFR was calculated using the Chronic Kidney Disease Epidemiology Collaboration (CKD-EPI) creatinine–cystatin C equation, at baseline and last follow-up.

eGFR decreased by 10–20% from baseline in 11/85 (14.1%) patients, 20–30% in 5/85 (5.9%), and ≥ 30% in 2/85 (2.4%) patients treated with ADV. Serum KIM-1 was more significantly increased after ADV treatment 86.53 (10.20–355.40) pg/mL than ETV treatment 61.54 (10.53–200.56) pg/mL (*P* < 0.01). Furthermore, serum KIM-1 was positively correlated with serum cystatin C (*r* = 0.47; *P* < 0.001) and negatively correlated with eGFR (*r* = -0.46; *P* < 0.001). The area under the receiver operating characteristic curve (AUC-ROC) of serum KIM-1 for identifying renal dysfunction in all enrolled patients was 0.94 (95% confidence interval [95% CI], 0.87 to 1.02; *P* < 0.001), while the AUC-ROC of serum creatinine was only 0.82 (95% CI, 0.60 to 1.03; *P* < 0.01).

Serum KIM-1 is a promising new diagnostic biomarker of renal dysfunction during long-term ADV therapy for CHB patients.

## Abstract 2

Supplemental Digital Content is available in the text

## 1 Introduction

Chronic hepatitis B virus (HBV) infection is a major public health problem, affecting more than 240 million people worldwide.^[1]^ Infected individuals are at an increased risk of developing liver cirrhosis, hepatic failure, and hepatocellular carcinoma (HCC), conditions that contribute to the high morbidity and mortality associated with HBV infection. Antiviral therapy can achieve sustained suppression of HBV replication and improve biochemical and histological indicators of liver disease, thereby slowing chronic hepatitis B (CHB) disease progression. Currently approved antiviral agents for CHB include interferon-alpha (IFN α) or pegylated interferon-alpha (PEG-IFN α) and nucleos(t)ide analogues (NUC). Although long-term use of oral NUC is associated with favorable efficacy and acceptable overall tolerance, drug resistance and side effects have been reported.

Adefovir dipivoxil (ADV) is an orally bioavailable prodrug of adefovir, an adenosine monophosphate analogue.^[2]^ It is now widely used as a rescue therapy in patients infected with viruses resistant to lamivudine (LAM), telbivudine, and entecavir (ETV).^[3,4]^ However, ADV is increasingly associated with nephrotoxicity. ADV nephrotoxicity is reported to occur in patients administered doses of 60 mg/d or 120 mg/d,^[5]^ whereas low doses of 10 mg/d are usually considered safe for the kidney.^[6,7]^ However, renal dysfunction was recently reported in HBV-infected patients administered 10 mg/d ADV.^[8,9]^ ADV-associated nephrotoxicity can cause increased serum creatinine, decreased estimated glomerular filtration rate (eGFR), hypophosphatemia, and Fanconi syndrome,^[9,10]^ and certain patients may be genetically predisposed to ADV-associated nephrotoxicity.^[11]^ However, routine renal function tests measuring serum creatinine, blood urea nitrogen (BUN), and eGFR, are likely to lack the sensitivity and specificity required for diagnosis of renal dysfunction and structural kidney injury in patients undergoing long-term ADV treatment. Therefore, identification of reliable and sensitive biomarkers of early renal dysfunction may facilitate early diagnosis, and improve the prognosis of CHB patients.

Kidney injury molecule-1 (KIM-1) is a type I transmembrane glycoprotein, highly expressed in epithelial cells in damaged regions of the renal proximal tubule. In both animal models and humans, urinary KIM-1 has been shown to be upregulated during acute kidney injury (AKI) caused by toxicity, ischemia, sepsis, and renal cell carcinoma.^[12]^ Elevations in urinary KIM-1 are significantly associated with gentamicin, cisplatin, vancomycin, and tacrolimus–induced kidney injury.^[13]^ Thus, KIM-1 has been qualified by the Food and Drug Administration (FDA) and European Medicines Agency (EMA) as a highly sensitive and specific urinary biomarker for monitoring drug-induced kidney injury.^[13,14]^ In addition to being present in urine, KIM-1 could also be released into the bloodstream as a result of altered microvascular permeability in humans, rats, and mice with AKI or chronic kidney disease (CKD).^[15]^ Circulating KIM-1 was recently reported to serve as a sensitive prognostic indicator in patients who were overdosed with acetaminophen.^[16]^ In this study, we aimed to investigate whether serum KIM-1 can be used as a reliable biomarker for the diagnosis of renal dysfunction in CHB patients receiving long-term ADV treatment.

## 2 Patients and methods

### 2.1 Patients

**Figure 1:**
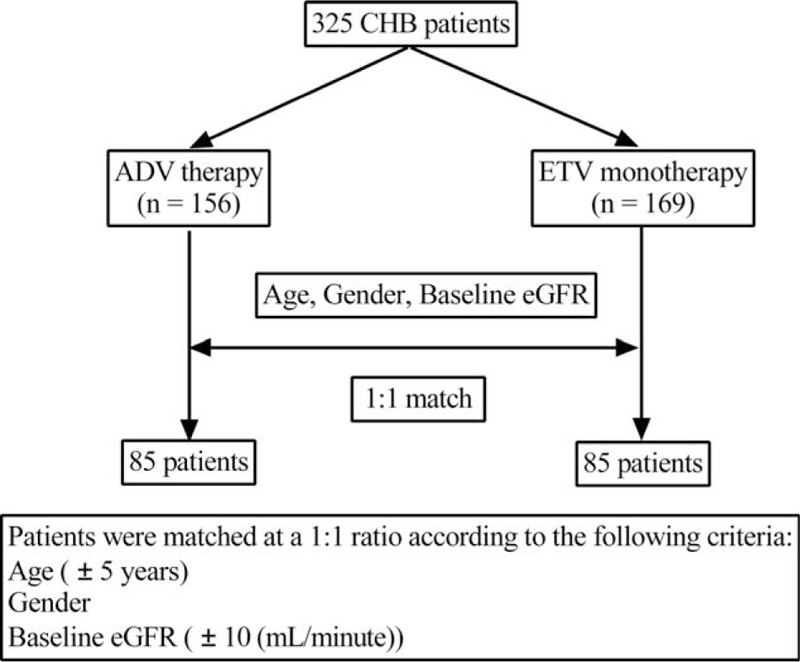
Flowchart of studied patients. ADV = adefovir dipivoxil, CHB = chronic hepatitis B, eGFR = estimated glomerular filtration rate, ETV = entecavir.

We performed a retrospective cohort study of 156 CHB patients treated for more than 6 months between January 2010 and August 2015 with 10 mg/d ADV in the Third Affiliated Hospital of Hebei Medical University and the Fifth Hospital of Shijiazhuang. An additional 169 CHB patients treated with ETV during the same period were selected as the comparison group. Both groups were matched for baseline age (± 5 years), sex, and eGFR classification (Fig. 1). The matched-pair platform consisted of 85 patients treated with ADV therapy and 85 patients with ETV monotherapy. All patients had a baseline eGFR ≥ 80 mL/min. Exclusion criteria were as follows: positivity for antibodies to human immunodeficiency virus or hepatitis C virus, diuretic users, hypertension, diabetes mellitus, HCC or other malignancy, autoimmune hepatitis, hepatic decompensation, alcoholic liver cirrhosis, severe heart and renal diseases, or the patients who take drugs that may affect the levels of KIM-1 (e.g., gentamicin, cisplatin, vancomycin, and tacrolimus).

The study protocol was approved by the Ethics Committee of the Third Affiliated Hospital of Hebei Medical University, and written informed consent was obtained from each subject during the screening period.

### 2.2 Study protocol

Patients visited our hospital every 3 months after the initiation of ADV or ETV treatment and routine virological and biochemical markers were measured. eGFR level was calculated using the Chronic Kidney Disease Epidemiology Collaboration (CKD-EPI) creatinine–cystatin C equation as follows: 135 × min (Scr/κ, 1)^α^ × max (Scr/κ, 1)^−0.601^ × min (Scys/0.8, 1)^−0.375^ × max (Scys/0.8, 1)^−0.711^ × 0.995 Age [× 0.969 if female] [× 1.08 if black], where Scr is serum creatinine, Scys is serum cystatin C, κ is 0.7 for females and 0.9 for males, α is −0.248 for females and −0.207 for males, min indicates the minimum of Scr/κ or 1, and max indicates the maximum of Scr/κ or 1.^[17]^ Serum cystatin C was measured by particle-enhanced nephelometric immunoassay. Renal impairment was classified as follows: unimpaired (eGFR ≥ 80 mL/min), mildly impaired (50 mL/min ≤ eGFR < 80 mL/min), moderately impaired (30 mL/min ≤ eGFR < 50 mL/min), and severely impaired (eGFR ≤ 30 mL/min).^[8]^

### 2.3 Serum KIM-1 quantification

Serum KIM-1 concentration was measured by enzyme-linked immunosorbent assay (ELISA) according to the manufacturer's instructions (RayBiotech, Norcross, GA). The detection range of the kit was 2–3000 pg/mL. Intra-assay and inter-assay precision were % coefficients of variation: <10% and <12%, respectively. The minimum detectable dose of KIM-1 was 2 pg/mL. Mean (95% confidence interval [CI]) plasma KIM-1 concentration in healthy volunteers was reported to be 64.4 (51.0–77.7) pg/mL.^[15]^

### 2.4 Statistical analysis

Data were analyzed using PRISM 6.0 (GraphPad Software, Inc., San Diego, CA) and SPSS 17.0 (SPSS, Chicago, IL). Descriptive statistics were reported as proportion (%) for categorical variables, and mean ± standard deviation or median (range) for continuous variables. Categorical variables were evaluated using the χ^2^ test. Normally distributed data were analyzed using Student's *t*-test. For non-normally distributed data, differences between groups were analyzed using Mann–Whitney *U* test. Bivariate correlation analysis was assessed by Spearman correlation test. Receiver operating characteristic curves (ROC) and the area under the ROC curve (AUC) were calculated to analyze the predictive value of the biomarkers. The Cox proportional hazard regression model was used to estimate univariate and multivariate risk factors for serum KIM-1 abnormality. Statistical significance was defined with a 2-tailed *P*-value < 0.05.

## 3 Results

### 3.1 Baseline clinical characteristics

A total of 85 CHB patients prescribed ADV and 85 CHB patients prescribed ETV were included in this retrospective cohort study. Baseline characteristics of the groups are presented in Table 1. The median duration of ADV therapy was 33 months (range: 8–66) and the median duration of ETV monotherapy was 34 months (range: 7–66). The ADV group and ETV group were well-matched with a similar baseline mean age, sex ratio, and eGFR classification.

**Table 1:**
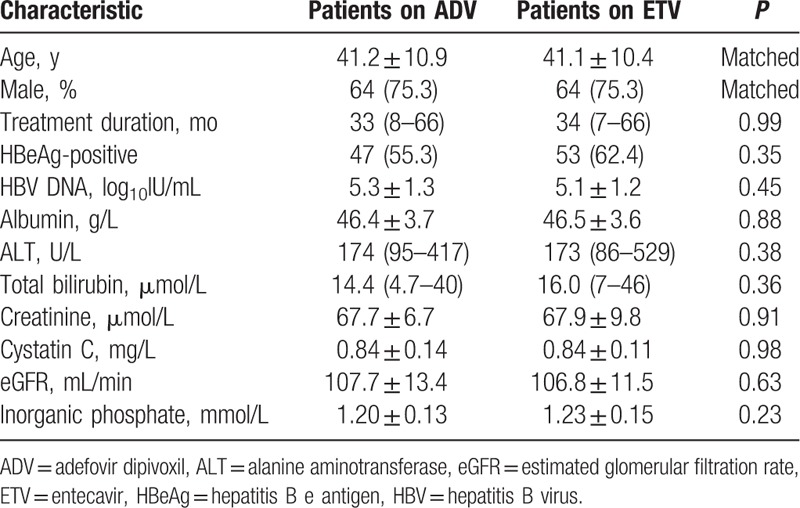
Baseline characteristics of all patients.

### 3.2 ADV-related nephrotoxicity

**Figure 2:**
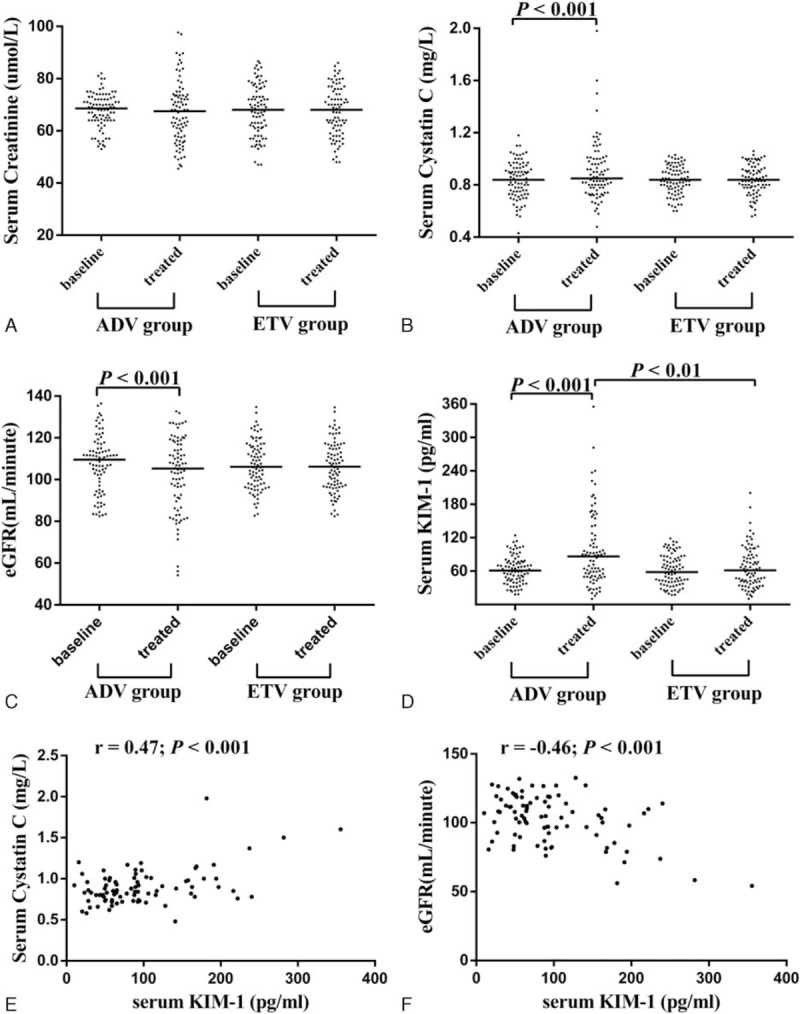
Evolution of various renal dysfunction indicators from baseline. Dot plots indicate serum creatinine (A), serum cystatin C (B), estimated glomerular filtration rate (eGFR) (C), and serum kidney injury molecule-1 (KIM-1) (D) for each patient. Scatterplot demonstrating a correlation between serum KIM-1 levels and serum cystatin C levels (*r* = 0.47; *P* < 0.001) (E), scatterplot demonstrating a correlation between serum KIM-1 levels and eGFR levels (*r* = -0.46; *P* < 0.001) (F).

The incidence and severity of decreased eGFR in the ADV group differed significantly from that in the ETV group. In the ADV group, eGFR decreased by 10–20% from baseline in 12/85 (14.1%) patients, 20–30% in 5/85 (5.9%), and ≥ 30% in 2/85 (2.4%) patients, and 8 patients (9.4%) developed mildly impaired renal function. In the ETV group, serum creatinine, serum cystatin C, and eGFR remained stable over the treatment period (Fig. 2A–C). Serum KIM-1 was significantly higher in the ADV group, 86.53 (10.20–355.40) pg/mL, than the ETV group, 61.54 (10.53–200.56) pg/mL (*P* < 0.01) (Fig. 2D). Serum KIM-1 was positively correlated with serum cystatin C (*r* = 0.47; *P* < 0.001) (Fig. 2E) and negatively correlated with eGFR (*r* = -0.46; *P* < 0.001) (Fig. 2F). In the ADV group, there were 37 patients treated with ADV monotherapy and 48 patients treated with ADV combination (Supplementary Table 1). There was no significant difference of kidney injury in the patients either treated with ADV alone or in combination with LAM (Supplementary Figure 1). Therefore, we did not probe further.

### 3.3 Diagnostic value of serum KIM-1

**Figure 3:**
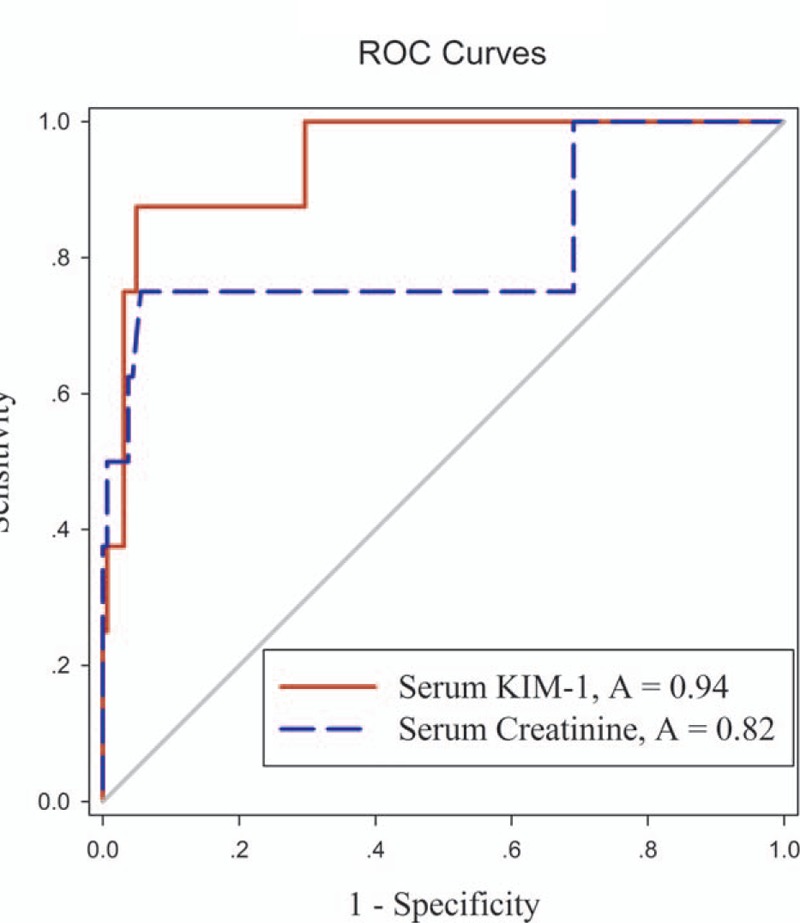
ROC curve analysis comparing performance of serum KIM-1 (solid red line, AUC 0.94) and serum creatinine (dashed blue line, AUC 0.82) levels. AUC = area under the ROC curve, KIM-1 = kidney injury molecule-1, ROC = receiver operating characteristic curve.

The AUC-ROC of serum KIM-1 for identifying renal dysfunction from all these populations, including both ADV and ETV treatment groups, was 0.94 (95% CI, 0.87 to 1.02; *P* < 0.001), while the AUC-ROC of serum creatinine was only 0.82 (95% CI, 0.60 to 1.03; *P* < 0.01) (Fig. 3). The cut-off of serum KIM-1 for diagnosis of renal dysfunction was 166.8 pg/mL, and sensitivity, specificity, positive predictive value, and negative predictive value were 88%, 95%, 47%, and 99%, respectively.

### 3.4 Predictive factors for serum KIM-1 abnormality

Serum levels of KIM-1 over 166.8 pg/mL were considered abnormal. Univariate analysis (Table 2) indicated that baseline eGFR < 90 mL/min (*P* = 0.005), and ADV treatment (*P* = 0.021) were significantly associated with the development of serum KIM-1 abnormality (*P* < 0.05). Multivariate analysis (Table 2) indicated that baseline eGFR < 90 mL/min (*P* = 0.025) was a significant predictor of serum KIM-1 abnormality.

**Table 2:**
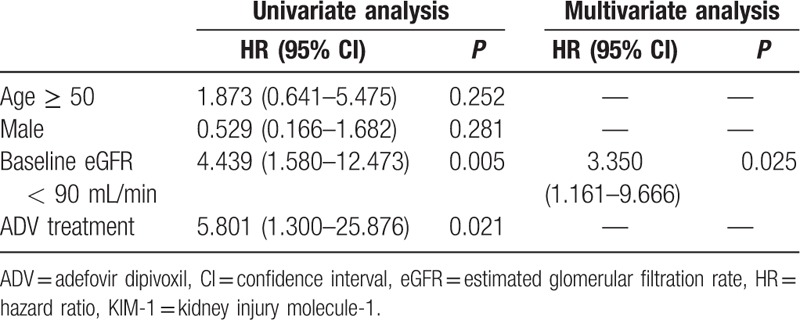
Determinants of serum KIM-1 abnormality.

## 4 Discussion

Although major international guidelines recommend both ETV and tenofovir as first-line therapies for CHB,^[18–20]^ ADV is widely used in some Asian countries due to the low cost and medical convention, as in the early 2000s ADV was the only alternative therapy for patients who developed LAM resistance. Accumulating evidence that ADV nephrotoxicity is dose-related has since caused the daily oral dose of ADV to be reduced from 120 mg to 10 mg.^[5,7,21]^

ADV is known to enter proximal kidney tubule cells through the basolateral human organic anion transporter 1 (hOAT1) and to be transported from tubule cells to the tubular lumen through multidrug resistance protein 2 (MRP2). As HBV DNA integrates into the human hepatocyte genomic DNA, long-term therapy is required, but extensive intracellular drug accumulation is associated with ADV nephrotoxicity.^[22]^ In a study of long-term therapy with ADV in 125 HBeAg-negative CHB patients for up to 5 years, 5% of patients experienced slight elevations in serum creatinine levels.^[23]^ In an open-label trial of long-term ADV therapy in 226 liver transplant candidates and 241 transplanted patients with LAM-resistant HBV infection, nephrotoxicity was observed in 6% and 21% of cases, respectively.^[24]^ In another retrospective study of 687 patients, over a median treatment duration of 27 months, 10.5% of patients developed renal impairment, defined by a decrease of >20% from baseline eGFR.^[25]^

In this study, we found that during a median of 33 months treatment with ADV, eGFR was decreased ≥ 10% from baseline in approximately 22.0% patients. Furthermore, only 8 patients developed adefovir-related mild renal dysfunction. The rates of eGFR depression are less pronounced than those reported previously,^[8,9,25]^ likely due to differences in patient number and ethnicity, in eGFR calculation methodology, and in the definition of renal dysfunction. Indeed, routine renal function was generally estimated by the rise in serum creatinine, a parameter known to be affected by many factors such as age, sex, race, muscle mass, metabolism, hydration status, nutrition status, medications. Recently, several urinary proteins such as β2-M, retinol-binding protein (RBP), neutrophil gelatinase-associated lipocalin (NGAL), and interleukin-18 (IL-18) have been evaluated as noninvasive indicators of kidney injury.^[26–28]^ However, these markers may not make ideal indicators as they can be unstable in the urine, modified as a result of urine physicochemical properties, or appear relatively late following kidney injury, and high-throughput methods for their detection are not well established.^[29]^ In addition, the consistency of their upregulation may vary between models of nephrotoxicity and upregulation may not be sustained throughout the time course of renal dysfunction, preventing accurate monitoring of injury progression and regression.^[29]^ KIM-1 is one of the most promising biomarkers for renal dysfunction due to the consistent results of preclinical and clinical trials. Measurement of KIM-1 is noninvasive, as KIM-1 is easily detectable in accessible body fluids (e.g., serum or urine), and highly sensitive and specific methods for detecting AKI rapidly and reliably may facilitate early detection of AKI and prediction of AKI severity and prognosis, unaffected by other biological variables.^[30]^

KIM-1 is a membrane receptor for human hepatitis A virus (HHAV) and T-cell immunoglobulin and mucin domain containing 4 (TIMD 4). KIM-1 is a single pass type I cell membrane glycoprotein that is expressed at a low level in normal kidney tissue but becomes highly expressed in dedifferentiated proximal tubule epithelial cells in human and rodent kidneys after ischemic or toxic injury.^[31,32]^

Since KIM-1 upregulation was detected in a rat model of renal ischemia, numerous animal and human studies have been performed in order to examine the diagnostic role of KIM-1 in AKI models. Ichimura et al^[29]^ examined tissue and urinary KIM-1 expression in a rat model of cisplatin-induced nephrotoxicity and found that KIM-1 is a faster and superior marker of nephrotoxicity than serum creatinine. In another rat study, the diagnostic value of urinary KIM-1 significantly exceeded traditional biomarkers (serum creatinine and BUN) as predictors of kidney tubular histopathological changes.^[33]^ In preclinical and clinical studies, urinary KIM-1 is reported to be a more sensitive diagnostic indicator of kidney injury than conventional biomarkers including serum creatinine, BUN, glycosuria, and increased proteinuria.^[13,34]^ Additional studies have confirmed that urinary KIM-1 concentration is upregulated in various kidney diseases including diabetic nephropathy, focal glomerulosclerosis, membranoproliferative glomerulonephritis, IgA nephropathy, and even renal cell carcinoma.^[35]^ Sabbisetti et al^[15]^ demonstrated that both in rodent and human AKI and mouse and human CKD, increased levels of KIM-1 can be detected in the blood, and KIM-1 serves as a biomarker of kidney injury.

We evaluated renal function using eGFR, calculated using prediction equations that take into account serum cystatin C and creatinine concentration as well as age, sex, and race. Cystatin C is a basic 13 kDa protein synthesized in all nucleated cells, and estimation of eGFR using cystatin C levels has clinical interest as it is not influenced by muscle mass, sex, or age.^[36]^ eGFR calculated by combining creatinine and cystatin C is reported to more accurately reflect measured GFR than either marker alone.^[17,37]^ Our results indicate that serum KIM-1 was correlated with serum cystatin C (*r* = 0.47; *P* < 0.001) and eGFR (*r* = -0.46; *P* < 0.001). Moreover, serum KIM-1 was significantly higher in the ADV group than the ETV group (*P* < 0.01). The superior diagnostic performance of serum KIM-1 was supported by AUC-ROC curve data.

We also analyzed risk factors for serum KIM-1 abnormality after long-term ADV treatment. Our univariate analysis indicated that baseline eGFR < 90 mL/min, and long-term ADV administration were significant and independent risk factors for serum KIM-1 abnormalities. Multivariate analysis also suggested that baseline eGFR < 90 mL/min was an independent risk factor for serum KIM-1 abnormality.

Our conclusions are, however, limited by the scope of this study. We enrolled a limited number of patients, did not perform continuous serum KIM-1 monitoring in CHB patients, and did not compare KIM-1 levels with other renal damage indicators. Despite its preliminary nature, this study clearly indicates that serum KIM-1 is a promising diagnostic biomarker for renal impairment during long-term adefovir therapy for CHB.

## Supplementary Material

Supplemental Digital Content
