# Supplementary material for: Circulating kidney injury molecule-1 is a novel diagnostic biomarker for renal dysfunction during long-term adefovir therapy in chronic hepatitis B
Source: Medicine (Baltimore). 2016 Nov 4;95(44):e5264. doi: 10.1097/MD.0000000000005264 (PMC5591140; doi:10.1097/MD.0000000000005264)
Supplement: Supplemental Digital Content [file medi-95-e5264-s001.doc]

**

**

Supplementary Figure 1 legend

Supplementary Figure 1 Evolution of various renal dysfunction indicators from baseline. Dot plots indicate Serum creatinine (A), serum cystatin C (B), estimated glomerular filtration rate (eGFR) (C) and serum KIM-1 (D) for patients treated with ADV.

**Supplementary Table 1** Baseline Characteristics For Patients On ADV

| **Baseline Characteristics** | [**Monotherapy**](javascript:void(0);)  (n=37) | [**Nombination**](javascript:void(0);) [**Therapy**](javascript:void(0);)  (n=48) | ***P* Value** |
| --- | --- | --- | --- |
| Age (yr) | 39.9±10.8 | 42.3±11.0 | 0.31 |
| Male (%) | 27(73.0) | 37(77.1) | 0.80 |
| Treatment duration (mo) | 33(8−65) | 33(10−66) | 0.53 |
| Creatinine (μmol/L) | 67.7±6.3 | 67.7±7.1 | 0.99 |
| Cystatin C (mg/L) | 0.82±0.13 | 0.85±0.15 | 0.39 |
| eGFR (mL/minute) | 109.1±11.3 | 106.7±14.9 | 0.42 |

ADV: Adefovir dipivoxil; eGFR: Estimated glomerular filtration rate.
